# Molecular Epidemiology and Antifungal Resistance of *Cryptococcus neoformans* From Human Immunodeficiency Virus-Negative and Human Immunodeficiency Virus-Positive Patients in Eastern China

**DOI:** 10.3389/fmicb.2022.942940

**Published:** 2022-07-05

**Authors:** Ziyi Zhou, Chendi Zhu, Margaret Ip, Manjiao Liu, Zhaoqin Zhu, Ryon Liu, Xiaomin Li, Lingbing Zeng, Wenjuan Wu

**Affiliations:** ^1^Department of Laboratory Medicine, Shanghai East Hospital, Tongji University School of Medicine, Shanghai, China; ^2^Department of Microbiology, The Chinese University of Hong Kong, Hong Kong, Hong Kong SAR, China; ^3^State Key Laboratory of Translational Medicine and Innovative Drug Development, Jiangsu Simcere Diagnostics Co., Ltd., Nanjing, China; ^4^Shanghai Public Health Clinical Center, Shanghai, China; ^5^Department of Laboratory Medicine, The First Affiliated Hospital of Nanchang University, Nanchang, China

**Keywords:** *Cryptococcus neoformans*, molecular epidemiology, antifungal susceptibility testing, resistance characteristics, whole genome sequencing

## Abstract

Cryptococcosis is an opportunistic and potentially lethal infection caused by *Cryptococcus neoformans* and *Cryptococcus gattii* complex, which affects both immunocompromised and immunocompetent people, and it has become a major public health concern worldwide. In this study, we characterized the molecular epidemiology and antifungal susceptibility of 133 *C. neoformans* isolates from East China Invasive Fungal Infection Group (ECIFIG), 2017–2020. Isolates were identified to species level by matrix-assisted laser desorption ionization-time of flight mass spectrometry and confirmed by *IGS1* sequencing. Whole-genome sequencing (WGS) was conducted on three multidrug-resistant isolates. Among the 133 strains, 61 (45.86%) were isolated from HIV-positive patients and 72 (54.16%) were isolated from HIV-negative patients. In total, *C. neoformans* var. *grubii* accounted for 97.74% (130/133), while *C. neoformans* var. *neoformans* was rare (2.06%, 3/133). The strains were further classified into nine sequence types (STs) dominated by ST5 (90.23%, 120/133) with low genetic diversity. No association was observed between STs and HIV status. All strains were wild type to voriconazole, while high antifungal minimal inhibitory concentrations (MICs) above the epidemiological cutoff values (ECVs) were observed in *C. neoformans* strains, and more than half of isolates were non-wild-type to amphotericin B (89.15%, 109/133). Eight isolates were resistant to fluconazole, and eight isolates were non-wild type to 5-fluorocytosine. Furthermore, WGS has verified the novel mutations of *FUR1* in 5-fluorocytosine-resistant strains. In one isolate, aneuploidy of chromosome 1 with G484S mutation of *ERG11* was observed, inducing high-level resistance (MIC: 32 μg/ml) to fluconazole. In general, our data showed that there was no significant difference between HIV-positive and HIV-negative patients on STs, and we elucidate the resistant mechanisms of *C. neoformans* from different perspectives. It is important for clinical therapy and drug usage in the future.

## Introduction

Cryptococcosis is one of the most common fungal diseases in the world, with an estimated 223,000 new cases and 181,100 deaths worldwide each year, primarily in southern Africa and Asia (Rajasingham et al., 2017). Cryptococcosis is an opportunistic and invasive fungal infection that not only has high rates of mortality and morbidity in immunocompromised or immunosuppression patients, like acquired immune deficiency syndrome (AIDS), but also infects immunocompetent individuals (Pyrgos et al., 2013; Sloan and Parris, 2014; Beardsley et al., 2019). There are mainly two species, namely, *Cryptococcus neoformans* and *Cryptococcus gattii*, with significant differences in ecology, molecular epidemiology, and antifungal sensitivity (Cogliati, 2013; Hagen et al., 2015; Firacative et al., 2021). In recent two decades, phylogenetic analysis based on genotypes and phenotypes has revealed two subtypes of *C. neoformans* and five subtypes of *C. gattii.* The major molecular types of *C. neoformans* have most commonly been designated molecular types VNI (AFLP1), VNII (AFLP1A/IB), and VNIII (AFLP3) for *C. neoformans* var. *grubii* and molecular types VNIV (AFLP2) for *C. neoformans* var. *neoformans* (Hagen et al., 2015, 2017; Kwon-Chung et al., 2017). Cryptococcosis is a more frequently observed fungal disease in AIDS patients in Europe, United States, and Africa (Dromer et al., 2007; Park et al., 2009; Pyrgos et al., 2013). The situation, however, is quite different in China. Previous studies showed that *C. neoformans* mainly originated from human immunodeficiency virus (HIV)-negative population without any risk factors reported in other countries (Feng et al., 2008; Khayhan et al., 2013).

The treatment strategies for cryptococcal meningitis recommended by the Infectious Diseases Society of America (IDSA) were amphotericin B plus 5-fluorocytosine for induction therapy and fluconazole used for consolidation therapy (Baddley and Forrest, 2019). However, it is easy to induce drug resistance for treating cryptococcosis due to the long-term and single therapeutic drug use (Bermas and Geddes-McAlister, 2020). According to a recent report by the China Invasive Fungi Surveillance Network, the cryptococcal resistance rate to fluconazole has increased more than threefold (10.5% in 2010 to 34% in 2014) (Xiao et al., 2018). In another multicenter study in China, the resistance rate of *C. neoformans* to fluconazole has dramatically risen, and non-wild-type isolates to 5-fluorocytosine have also been found (Fan et al., 2016).

So far, the resistance mechanisms of *C. neoformans* were understudied. According to previous studies, cryptococcal resistance to fluconazole could be caused by point mutations of *ERG11* (G1785C, G1855A, and G1855T) (Rodero et al., 2003; Bosco-Borgeat et al., 2016; Gago et al., 2017; Selb et al., 2019), overexpression of *ERG11*, overexpression of *AFR1*, and aneuploidy formation. Acquisition of aneuploidies in *C. neoformans* can mediate increased MIC values to fluconazole and further enable cross-adaptation to other antifungal drugs (Yang F. et al., 2021). Mutations of *FCY1*, *FCY2*, and *FUR1* were the most common 5-fluorocytosine resistance mechanism of cryptococcus (Vu et al., 2018). Recent studies have demonstrated that mutations of *UXS1* are also involved with 5-fluorocytosine resistance (Billmyre et al., 2020; Chang et al., 2021). Indeed, comprehensive genomic characterization of *C. neoformans* is limited in China. Notably, antifungal susceptibility, particularly to fluconazole and 5-fluorocytosine, has been noted to vary in correlation not only with molecular types but also with HIV status (Espinel-Ingroff et al., 2012; Li et al., 2012; Arsic Arsenijevic et al., 2014). To investigate the molecular epidemiology of local cryptococcal isolates, several molecular typing methods have been developed, for example, PCR-fingerprinting, randomly amplified polymorphic DNA (RAPD), PCR-restriction fragment length polymorphism (PCR-RFLP), amplified fragment length polymorphism (AFLP), microsatellite typing, multilocus microsatellite typing (MLMT), multilocus sequence typing (MLST), and whole-genome sequencing (WGS) (Bovers et al., 2008; Meyer et al., 2009; Li et al., 2013; Hong et al., 2021). Extensive studies have recommended MLST as the preferred method among these molecular techniques because of its excellent discrimination ability and reproducibility between different laboratories. A normative MLST scheme of the *C. neoformans*/*C. gattii* has been established by the International Society of Human and Animal Mycoses (ISHAM) working group (Meyer et al., 2009). Seven housekeeping genes (*CAP59*, *GPD1*, *IGS1*, *LAC1*, *PLB1*, *SOD1*, and *URA5*) were selected for MLST analysis of the *C. neoformans*/*C. gattii*^1^ ^,2^, and WGS exhibited high reproducibility, specificity, and discriminating power. Therefore, in this study, we explore the prevalence and antifungal drug resistance mechanism of *C. neoformans* in HIV-positive and HIV-negative patients in China by using high-precision MLST and WGS.

## Materials and Methods

### Clinical Isolates Information

Exactly 133 cryptococcal isolates were collected from East China Invasive Fungal Infection Group (ECIFIG) between 2017 and 2020. Sixty-one isolates derived from HIV-infected patients who had HIV antibody screening test and confirmatory tests positive were classified as HIV-positive group, while others were classified as HIV-negative group. All isolates were identified to species level by matrix-assisted laser desorption/ionization time-of-flight mass-spectrometry (Zybio, China) and confirmed by *IGS1* sequencing. Ethics approval (2021-061) for this study was obtained from the Health Research Ethics Board of Shanghai East Hospital.

### Antifungal Susceptibility Testing

We conducted the antifungal susceptibility testing of 133 isolates against amphotericin B (AMB), 5-fluorocytosine (5FC), fluconazole (FCZ), and voriconazole (VCZ) by using the broth microdilution method (BMD) according to the CLSI M27-A4 guidelines (CLSI, 2017). In brief, isolates were sub-cultured on Sabouraud’s dextrose agar (SDA) (Oxoid, United Kingdom) at 35°C for 48 h, the suspension was adjusted by McFarland in a sterile solution, and then, antifungal susceptibility tests were performed. *Candida krusei* ATCC 6258 and *Candida parapsilosis* ATCC 22019 were used as quality control. Epidemiological cutoff values (ECVs) were used to determine wild-type and non-wild-type strains of some antifungals due to lack of breakpoint. ECVs were recommended by CLSI M59: AMB, 0.5 μg/ml (VNI); 5FC, 8 μg/ml (VNI); FCZ, 8 μg/ml (VNI); and VCZ, 0.25 μg/ml (VNI) (CLSI, 2018).

### DNA Extraction

DNA extraction of isolates was performed by the method described by Xu et al. (2000) with some modifications. Briefly, all the isolates were sub-cultured on SDA at 30°C for 48–72 h. Monoclonal colonies were collected in the sterile Eppendorf (EP) tubes containing 50 mg glass beads (BioSpec, United States), 200 μl lysis buffer, 200 μl phenol-chloroform, and broken for 10 min, and then centrifuged at high speed for 5 min. Supernatants were transported to new EP tubes. DNAs were extracted by phenol-chloroform alcohol and stored at −20°C.

### Intergenic Spacer 1 Sequencing and Multilocus Sequence Typing Analysis

Identification of *Cryptococcus* spp. through amplification of the intergenic spacer 1 (*IGS1*) region was amplified using primers, *IGS1*F (5′-TAAGCCCTTGTT-3′) and *IGS1*R (5′-AAAGATTTATTG-3′), from ISHAM (see text footnote 2). Polymerase chain reaction (PCR) of the *IGS1* gene was performed in a 30 μl final volume. The PCR mixture contains 1 μl of DNA, 15 μl of PCR enzyme mix, and 1 μl of each primer. For PCR amplification, the PCR mixture was denatured for 5 min at 94°C followed by 35 cycles of 30 s at 94°C, 30 s at 53°C, and 1 min at 72°C, followed by one final step of 10 min at 72°C. For MLST analysis, PCR was performed on seven housekeeping genes (*CAP59*, *GPD1*, *IGS1*, *LAC1*, *PLB1*, *SOD1*, and *URA5*) according to the International Fungal Multi Locus Sequence Typing Database (IFMLST) (see text footnote 2). Each PCR system was amplified in a 30 μl final volume as described before, the reaction procedure was described in the IFMLST profile, and all the primers were listed in the IFMLST. Then, all PCR products were purified with Gel Extraction Kit 200 (Omega Bio-Tek, United States) according to the manufacturer’s instructions and were sequenced by an ABI 3730XL DNA analyzer (Shanghai, China). Sequences were assigned to the IFMLST consensus MLST scheme database to obtain sequence types (STs).

### Whole-Genome Sequencing

Three multidrug resistance isolates with MIC ≥16 μg/ml to FCZ and 5-FC were selected for whole-genome sequencing (WGS) in this study. Among them, one isolate was separated from the HIV-positive group (YQJ185), and the other two isolates were separated from the HIV-negative group (YQJ68 and YQJ247). All isolates were sub-cultured on SDA at 35°C for 48 h according to the CLSI M27-A4, and then, DNA was extracted using Zymo Quick-DNA/RNA Viral Kit (D7020), followed by library preparation using Vazyme transposase-based approach (TD502). WGS was performed using Illumina NovaSeq 6000 platform.

### Bioinformatics

Raw reads were quality-controlled and trimmed with Trimmomatic (Bolger et al., 2014). SPAdes were applied for short-read assembly (Bankevich et al., 2012). The YMAP pipeline was used for mapping with reference genome H99 and computing depth to estimate the variation of copy numbers and ploidy across chromosomes (Abbey et al., 2014). To determine the MAT type, short-read sequences were aligned to MATa locus (AF542528) and MAT α (alpha) locus (AF542529).

Reads were aligned to the H99 reference genome (Janbon et al., 2014) using BWA-MEM (Li and Durbin, 2009). Alignments were further processed with SAMtools (Li et al., 2009) and Genome Analysis Toolkit (GATK) (McKenna et al., 2010). SNP and indel calling were performed using the HaplotypeCaller Component of the GATK with default settings. Variants were further filtered with filter expression “QUAL < 30.0 || QD < 2.0 || FS > 60.0 || SOR > 4.0” using VariantFiltration Component of GATK. Variants were annotated using SnpEff (Cingolani et al., 2012) and FungiDB (Stajich et al., 2012). Candidate fungi resistance-related variants were collected from publications [*ERG11* (CNAG_00040), *UXS1* (CNAG_03322), *FUR1* (CNAG_02337), *FCY1* (CNAG_00613), *FCY2* (CNAG_01681), and MSH2 (CNAG_00770)]. All candidate resistance-related variant calls were visually examined using the Integrated Genome Viewer (IGV) to remove calls resulting from poor read mapping (Thorvaldsdóttir et al., 2013). Global ST5 isolates from previous studies were retrieved from National Center for Biotechnology Information (NCBI) (Rhodes et al., 2017; Ashton et al., 2019). Core SNP phylogenetic tree was generated using IQTREE with H99 as outgroup and 10000 Ultrafast Bootstrap to support branch (Nguyen et al., 2015).

### Statistical Analysis

Categorized variables were analyzed by Fisher’s exact test by IBM SPSS software (version 26.0). Continuous variables were calculated by Mann–Whitney *U* test. A *p* < 0.05 was considered significant.

## Results

### Antifungal Susceptibility Test

**FIGURE 1:**
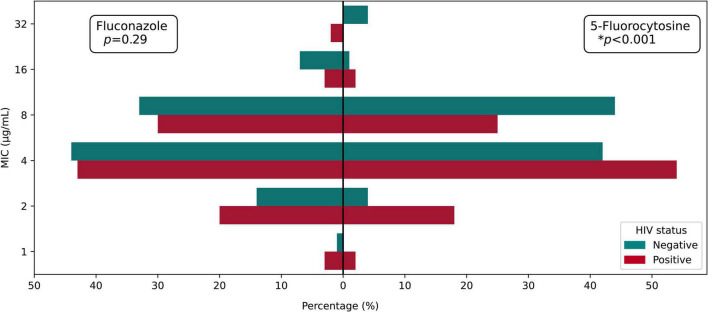
MIC distribution of fluconazole and 5-fluorocytosine among HIV-positive and HIV-negative groups.

*In vitro* antifungal susceptibility testing of total isolates was performed against four agents. In brief, the majority exhibited high sensitivity to fluconazole, 5-fluorocytosine, and voriconazole, ranging from 93.98 to 100%. However, 89.15% (109/133) of isolates were non-wild type to amphotericin B. Eight isolates were resistant to fluconazole, and eight isolates were non-wild type against 5-fluorocytosine; compared with the recommended ECVs of fluconazole and 5-fluorocytosine, high MICs of cryptococcal isolates against 5-fluorocytosine (64 μg/ml) or fluconazole (32 μg/ml) were observed. Interestingly, we found three multidrug isolates (1 isolate from HIV-positive group and 2 isolates from HIV-negative group) (Table 1 and Supplementary Table 1). For isolates from HIV-positive and HIV-negative groups, the MIC distribution was similar in fluconazole (*p* = 0.290) but significantly different in 5-fluorocytosine (*p* < 0.001), with higher MIC values in HIV-negative group (Figure 1).

**TABLE 1:** *In vitro* susceptibilities of *Cryptococcus neoformans* in HIV-positive and HIV-negative cryptococcosis patients.

HIV status	Species (No. of isolates)	Antifungal drugs	MIC (μg/mL)						
			Range	MIC_50_	MIC_90_	GM	Mode MIC	WT%	Non-WT%
HIV-positive	*Cryptococcus neoformans* (*n* = 61)	Fluconazole	1–32	4	8	4.43	4	95.08	4.92
		Voriconazole	0.03125–0.25	0.0625	0.125	0.08	0.0625	100	0
		Amphotericin B	0.25–2	1	2	1.23	1	9.84	90.16
		Flucytosine	1–16	4	8	4.19	4	98.36	1.64
HIV-negative	*Cryptococcus neoformans* (*n* = 72)	Fluconazole	1–16	4	8	4.94	4	93.06	6.94
		Voriconazole	0.015–0.25	0.0625	0.125	0.08	0.0625	100	0
		Amphotericin B	0.0125–2	1	2	1.01	1	25	75
		Flucytosine	2–64	8	16	6.59	8	90.28	9.72

*MIC, minimum inhibitory concentration; MIC50 and MIC90, MICs at which 50 and 90% of isolates were inhibited; GM, geometric mean; WT, wild type; NWT, non-wild type.*

### Identification and Correlation Between ST5 and Human Immunodeficiency Virus Status

According to MALDI-TOF MS and *IGS1* sequencing outcomes, the 133 *C. neoformans* clinical isolates included 130 *C. neoformans* var. *grubii* and 3 *C. neoformans* var. *neoformans*. Among the 3 *C. neoformans* var. *neoformans* isolates, two isolates were from the HIV-positive group (ST77 and ST93), and the remaining isolate was from the HIV-negative group (ST185). As for MLST analysis, in this study, all isolates were classified into nine STs, and the majority of isolates belonged to ST5, VNI (90.23%, 120/133). For HIV-positive group, ST5 accounted for 86.88% (53/61), and five isolates with other STs included ST43 (1.64%), ST63 (1.64%), ST77 (1.64%), ST93 (1.64%), and ST230 (1.64%). In the other group, there were four STs, containing ST5 (93.05%, 67/72), ST31 (1.39%), ST185 (1.39%), and ST653 (2.78%). In comparison with the HIV-negative group, STs of the HIV-positive group exhibited more diversity. There were four isolates unknown to STs due to failure of sequencing or identifying. In addition, compared with the HIV-positive group, there was no correlation between HIV status and STs (*p* = 0.256). More details are provided in Supplementary Tables 2, 3. In general, our study revealed that *C. neoformans* var. *grubii* (ST5, VNI) was the most representative and predominant species in East China.

### Whole-Genome Sequencing

**FIGURE 2:**
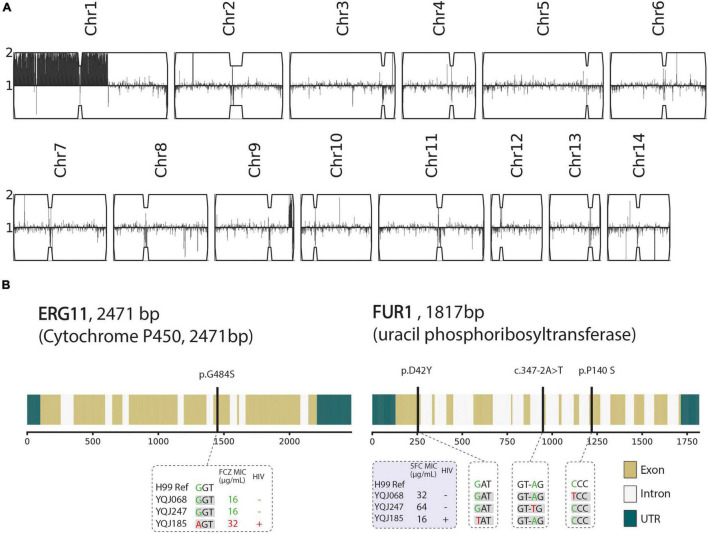
Genetic characteristics of three resistant strains. **(A)** Karyotype of multidrug-resistant strain (YQJ185). Copy number is labeled in *y*-axis compared with haploid H99 reference using YMAP, with 1 copy number as baseline. Position of reads of each chromosome is on *x*-axis. **(B)** Non-synonymous mutation and splice site mutation of *ERG11* and *FUR1* genes.

In this study, we analyzed the mating type and resistant mechanisms from three multidrug-resistant strains by WGS. The detailed information about WGS, including total reads, base quality, depth, and coverage, is shown in Supplementary Table 4. All multidrug-resistant strains belonged to MAT α. For an isolate (YQJ185) from the HIV-positive group, aneuploidy occurred in chromosome 1, but not in other chromosomes (Figure 2A). G484S mutation was found in *ERG11* gene of YQJ185 with a high-level MIC (32 μg/ml) to FCZ, located in the conserved heme-binding domain. Copy number variant (CNV) and *ERG11* mutation, however, were not observed in the other two resistant isolates from the HIV-negative group. The non-synonymous mutation was also observed in *FUR1* in different positions. For the HIV-positive group, D42Y mutation was found in the *FUR1* gene of YQJ185 with MIC (16 μg/ml) to 5FC. For the HIV-negative group, P140S mutation was found in the *FUR1* gene of YQJ68 with a high MIC (32 μg/ml) to 5FC, while YQJ247 has an A-T transition in an intron splice site (Figure 2B).

**FIGURE 3:**
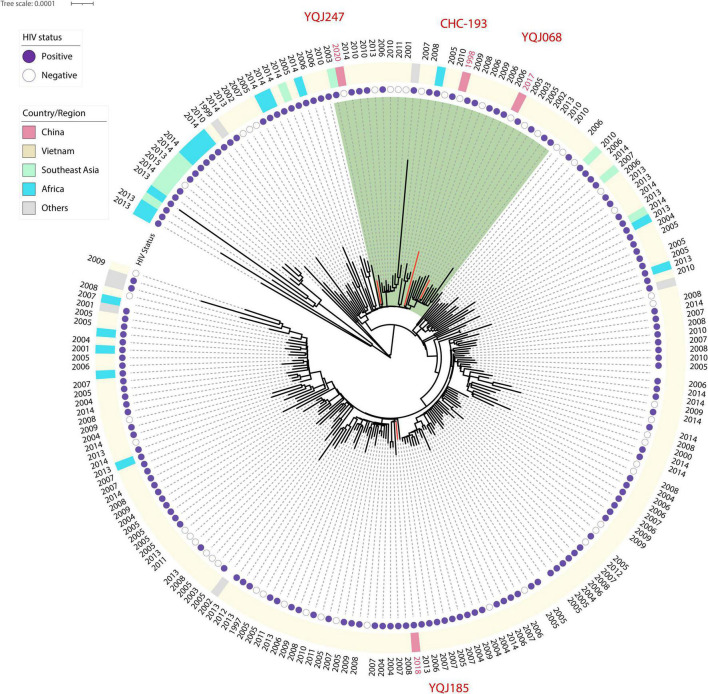
Core SNP phylogenetic tree of global ST5 *C. neoformans*. H99 reference was used as reference and outgroup; 44,487 SNP sites were used to generate this tree. Red branches represent isolates from China.

The ST5 isolates were located in the subclade of VNIa. As mentioned above, ST5 is the major genotype in China, but whole-genome sequences were rarely published. Phylogenetic relationships including ST5 isolates from other countries were generated in this study. Two isolates from HIV-negative patients were clustered into the same subclade with CHC-193 isolated from an HIV-negative patient in 1998 in China (Figure 3).

## Discussion

*Cryptococcus neoformans* is widely distributed in the world, and usually, it infects HIV-positive patients, particularly in South Africa and Asia (Rajasingham et al., 2017). However, the condition appears to be extremely different in China (Chen et al., 2018). Previous studies showed that cryptococcosis was likely to occur in immunocompetent individuals or in individuals with other underlying diseases (Fang et al., 2020; Li et al., 2020). Indeed, *C. neoformans* exhibited lower genetic diversity in China than that in South Asia, and ST5 was the predominant genotype (Khayhan et al., 2013; Dou et al., 2015; Thanh et al., 2018).

The MLST was one of the most common technologies to analyze the genotypic diversity of *C. neoformans*. In this study, our results showed that there was lower genetic diversity of *C. neoformans*, and ST5 is the dominant ST in China, accounting for 90.23% (120/133) in total. The same results were observed in previous Chinese studies (Chen et al., 2018; Yang C. et al., 2021). Indeed, our research revealed no significant difference between HIV-positive and HIV-negative patients on STs (*p* = 0.256). This is consistent with southwest China (Wu et al., 2021). However, the situation is different in South Korea, where there were significant differences between HIV status and genetic types (Choi et al., 2010). In another study from Asia, it was affirmed that most isolates from HIV-negative patients were ST5 (Khayhan et al., 2013). Furthermore, in this study we identified five new STs in China, namely, ST230, ST43, ST77, ST185, and ST653, and all of the STs haven’t been reported yet in East China (Yang C. et al., 2021). Most importantly, ST31 was the most common ST for environmental *C. neoformans* in China, which mainly originated from pigeon droppings (Dou et al., 2017; Chen et al., 2021), and ST31 was also the main ST of *C. neoformans* in India (Xess et al., 2021). This suggests that attention is paid to the clinical isolates of *C. neoformans* but environmental isolates of *C. neoformans* need to be investigated more deeply and more extensively in the future.

Fluconazole and amphotericin B are the most frequent therapeutic drugs in cryptococcosis treatment. High MICs of amphotericin B above ECVs are concerned in this study, while all isolates were sensitive to voriconazole. This is consistent with a 6-year retrospective study from Hunan, China (Li et al., 2020). Interestingly, the MIC distribution of 5-fluorocytosine in the HIV-negative group was higher than that of the HIV-positive group, and there were no significant differences in other drugs. This is opposite to the study in Southeast China and is consistent with the study in Serbia (Li et al., 2012; Arsic Arsenijevic et al., 2014). Moreover, in a study from southeast China, the results exhibited no significant differences in antifungal susceptibility to fluconazole and 5-fluorocytosine between HIV-positive and HIV-negative patients (Wu et al., 2021). However, the association between STs and antifungal susceptibility was not observed. In this study, three multidrug isolates were found. Therefore, in this study, we investigated the resistance mechanisms through WGS. Aneuploidy of Chromosome 1 of an isolate (YQJ185) from an HIV-infected patient was tested. Previous studies proved the correlation between the formation of aneuploidy of Chromosome 1 and excessive doses of fluconazole (Stone et al., 2019; Yang F. et al., 2021). In addition, we also revealed a point mutation of *ERG11* (G484S) (Rodero et al., 2003; Gago et al., 2017). CNV and *ERG11* mutation would accelerate the speed of the cryptococcal resistance to fluconazole. However, the same resistance mechanisms were not observed in the other two isolates (HIV-negative group) against fluconazole (16 μg/ml). What’more, there was no available literature that described the resistance mechanisms of 5-fluorocytosine in China. In this study, we addressed point mutations of *FUR1* in different mutation sites and splice site mutation with different MICs, and it exhibited unique resistance mechanisms of 5-fluorocytosine in China. Previous studies reported that genes *FCY1*, *FCY2*, and *UXS1* were associated with resistance to 5-fluorocytosine (Vu et al., 2018; Billmyre et al., 2020); however, we didn’t find it in our study. ST5 or VNIa-5 is an important phylogenetic group in Southeast Asia, characterized by its ability to infect HIV-negative patients (Ashton et al., 2019). Despite all three genomes in this study were closely related to Vietnam strains, they are assigned to two subclades, indicating the unique evolution progress of the strain from the HIV-positive group.

## Conclusion

Cryptococcus in China exhibited a low extent of genetic diversity, whether HIV-positive or HIV-negative patients were not linked to STs. VNI is the dominant molecular type in *C. neoformans* and ST5 is the predominant ST. Phylogenetic relationship and resistance mechanisms have evolved among the subclades of ST5 isolates with certain particularity in China. However, there are limitations in this study. First, the geographical representativeness of epidemiological characteristics and resistance mechanisms in this study is limited, only representing East China. Second, only three clinical isolates were performed by WGS in our study, and the correlation among clinical isolates, standard isolates, and environmental isolates should be involved in the future. Finally, the isolates should be inoculated on the medium with FCZ, which would contribute to finding a resistance mechanism on the genomic level. WGS can be used to discover more than just about evolutionary relationships. Hence, we are taking steps to establish a database of cryptococcal genomes using WGS in East China.

## Data Availability Statement

The datasets presented in this study can be found in online repositories. The names of the repository/repositories and accession number(s) can be found below: https://ngdc.cncb.ac.cn/bioproject/browse/PRJCA009353, PRJCA009353.

## Author Contributions

WW designed the experiments and supervised the data analysis. ZYZ and CZ wrote the manuscript. CZ, ML, RL, and XL performed and interpreted the whole-genome sequencing data. WW, LZ, ZQZ, and ZYZ collected the strains. All authors contributed to the collection and assembly of data, manuscript writing, and final approval of the manuscript.

## Conflict of Interest

ML, RL, and XL were employed by the Jiangsu Simcere Diagnostics Co., Ltd. The remaining authors declare that the research was conducted in the absence of any commercial or financial relationships that could be construed as a potential conflict of interest.

## Publisher’s Note

All claims expressed in this article are solely those of the authors and do not necessarily represent those of their affiliated organizations, or those of the publisher, the editors and the reviewers. Any product that may be evaluated in this article, or claim that may be made by its manufacturer, is not guaranteed or endorsed by the publisher.

## Footnotes

1
http://www.mlst.net/

2
http://mlst.mycologylab.org

## References

[B1] Abbey D. A. Funt J. Lurie-Weinberger M. N. Thompson D. A. Regev A. Myers C. L. (2014). YMAP: a pipeline for visualization of copy number variation and loss of heterozygosity in eukaryotic pathogens. *Genome Med.* 6:100. 10.1186/s13073-014-0100-8 25505934 PMC4263066

[B2] Arsic Arsenijevic V. Pekmezovic M. G. Meis J. F. Hagen F. (2014). Molecular epidemiology and antifungal susceptibility of Serbian Cryptococcus neoformans isolates. *Mycoses* 57 380–387. 10.1111/myc.12171 24438323

[B3] Ashton P. M. Thanh L. T. Trieu P. H. Van Anh D. Trinh N. M. Beardsley J. (2019). Three phylogenetic groups have driven the recent population expansion of Cryptococcus neoformans. *Nat. Commun.* 10:2035. 10.1038/s41467-019-10092-5 31048698 PMC6497710

[B4] Baddley J. W. Forrest G. N. (2019). Cryptococcosis in solid organ transplantation-Guidelines from the American Society of Transplantation Infectious Diseases Community of Practice. *Clin. Trans.* 33:e13543. 10.1111/ctr.13543 30900315

[B5] Bankevich A. Nurk S. Antipov D. Gurevich A. A. Dvorkin M. Kulikov A. S. (2012). SPAdes: a new genome assembly algorithm and its applications to single-cell sequencing. *J. Comput. Biol.* 19 455–477. 10.1089/cmb.2012.0021 22506599 PMC3342519

[B6] Beardsley J. Sorrell T. C. Chen S. C. (2019). Central Nervous System Cryptococcal Infections in Non-HIV Infected Patients. *J. Fungi* 5:71. 10.3390/jof5030071 31382367 PMC6787755

[B7] Bermas A. Geddes-McAlister J. (2020). Combatting the evolution of antifungal resistance in Cryptococcus neoformans. *Mol. Microbiol.* 114 721–734. 10.1111/mmi.14565 32697029

[B8] Billmyre R. B. Applen Clancey S. Li L. X. Doering T. L. Heitman J. (2020). 5-fluorocytosine resistance is associated with hypermutation and alterations in capsule biosynthesis in Cryptococcus. *Nat. Commun.* 11:127. 10.1038/s41467-019-13890-z 31913284 PMC6949227

[B9] Bolger A. M. Lohse M. Usadel B. (2014). Trimmomatic: a flexible trimmer for Illumina sequence data. *Bioinformatics* 30 2114–2120. 10.1093/bioinformatics/btu170 24695404 PMC4103590

[B10] Bosco-Borgeat M. E. Mazza M. Taverna C. G. Córdoba S. Murisengo O. A. Vivot W. (2016). Amino acid substitution in Cryptococcus neoformans lanosterol 14-α-demethylase involved in fluconazole resistance in clinical isolates. *Rev. Argent. Microbiol.* 48 137–142. 10.1016/j.ram.2016.03.003 27311753

[B11] Bovers M. Hagen F. Kuramae E. E. Boekhout T. (2008). Six monophyletic lineages identified within Cryptococcus neoformans and Cryptococcus gattii by multi-locus sequence typing. *Fungal Genet. Biol.* 45 400–421. 10.1016/j.fgb.2007.12.004 18261945

[B12] Chang Y. C. Lamichhane A. K. Cai H. Walter P. J. Bennett J. E. Kwon-Chung K. J. (2021). Moderate levels of 5-fluorocytosine cause the emergence of high frequency resistance in cryptococci. *Nat. Commun.* 12:3418. 10.1038/s41467-021-23745-1 34103502 PMC8187385

[B13] Chen M. Wang Y. Li Y. Hong N. Zhu X. Pan W. (2021). Genotypic diversity and antifungal susceptibility of environmental isolates of Cryptococcus neoformans from the Yangtze River Delta region of East China. *Med. Mycol.* 59 653–663. 10.1093/mmy/myaa096 33269400

[B14] Chen M. Xu Y. Hong N. Yang Y. Lei W. Du L. (2018). Epidemiology of fungal infections in China. *Front. Med.* 12 58–75. 10.1007/s11684-017-0601-0 29380297

[B15] Choi Y. H. Ngamskulrungroj P. Varma A. Sionov E. Hwang S. M. Carriconde F. (2010). Prevalence of the VNIc genotype of Cryptococcus neoformans in non-HIV-associated cryptococcosis in the Republic of Korea. *FEMS Yeast Res.* 10 769–778. 10.1111/j.1567-1364.2010.00648.x 20561059 PMC2920376

[B16] Cingolani P. Platts A. Wang le L. Coon M. Nguyen T. Wang L. (2012). A program for annotating and predicting the effects of single nucleotide polymorphisms, SnpEff: SNPs in the genome of Drosophila melanogaster strain w1118; iso-2; iso-3. *Fly.* 6 80–92. 10.4161/fly.19695 22728672 PMC3679285

[B17] CLSI (2017). *Clinical and Laboratory Standards Institute (2017. b). Reference Method for Broth Dilution Antifungal Susceptibility Testing of Yeasts, 4th Ed.* Wayne, PA: CLSI.

[B18] CLSI (2018). *Epidemiological Cutoff Values for Antifungal Susceptibility Testing, 2nd Edn.* Wayne, PA: CLSI.

[B19] Cogliati M. (2013). Global Molecular Epidemiology of Cryptococcus neoformans and Cryptococcus gattii: An Atlas of the Molecular Types. *Scientifica* 2013:675213. 10.1155/2013/675213 24278784 PMC3820360

[B20] Dou H. Wang H. Xie S. Chen X. Xu Z. Xu Y. (2017). Molecular characterization of Cryptococcus neoformans isolated from the environment in Beijing. *China. Med. Mycol.* 55 737–747. 10.1093/mmy/myx026 28431114

[B21] Dou H. T. Xu Y. C. Wang H. Z. Li T. S. (2015). Molecular epidemiology of Cryptococcus neoformans and Cryptococcus gattii in China between 2007 and 2013 using multilocus sequence typing and the DiversiLab system. *Eur. J. Clin. Microbiol. Infect. Dis.* 34 753–762. 10.1007/s10096-014-2289-2 25471194

[B22] Dromer F. Mathoulin-Pélissier S. Launay O. Lortholary O. (2007). Determinants of disease presentation and outcome during cryptococcosis: the CryptoA/D study. *PLoS Med.* 4:e21. 10.1371/journal.pmed.0040021 17284154 PMC1808080

[B23] Espinel-Ingroff A. Aller A. I. Canton E. Castañón-Olivares L. R. Chowdhary A. Cordoba S. (2012). Cryptococcus neoformans-Cryptococcus gattii species complex: an international study of wild-type susceptibility endpoint distributions and epidemiological cutoff values for fluconazole, itraconazole, posaconazole, and voriconazole. *Antimicrob. Agents Chemother.* 56 5898–5906. 10.1128/aac.01115-12 22948877 PMC3486550

[B24] Fan X. Xiao M. Chen S. Kong F. Dou H. T. Wang H. (2016). Predominance of Cryptococcus neoformans var. grubii multilocus sequence type 5 and emergence of isolates with non-wild-type minimum inhibitory concentrations to fluconazole: a multi-centre study in China. *Clin. Microbiol. Infect.* 22:887.e1-887.e9. 10.1016/j.cmi.2016.07.008 27432767

[B25] Fang L. F. Zhang P. P. Wang J. Yang Q. Qu T. T. (2020). Clinical and microbiological characteristics of cryptococcosis at an university hospital in China from 2013 to 2017. *Braz. J. Infect. Dis.* 24 7–12. 10.1016/j.bjid.2019.11.004 31870760 PMC9392018

[B26] Feng X. Yao Z. Ren D. Liao W. Wu J. (2008). Genotype and mating type analysis of Cryptococcus neoformans and Cryptococcus gattii isolates from China that mainly originated from non-HIV-infected patients. *FEMS Yeast Res.* 8 930–938. 10.1111/j.1567-1364.2008.00422.x 18671745

[B27] Firacative C. Meyer W. Castañeda E. (2021). Cryptococcus neoformans and Cryptococcus gattii Species Complexes in Latin America: A Map of Molecular Types, Genotypic Diversity, and Antifungal Susceptibility as Reported by the Latin American Cryptococcal Study Group. *J. Fungi.* 7:282. 10.3390/jof7040282 33918572 PMC8069395

[B28] Gago S. Serrano C. Alastruey-Izquierdo A. Cuesta I. Martin-Mazuelos E. Aller A. I. (2017). Molecular identification, antifungal resistance and virulence of Cryptococcus neoformans and Cryptococcus deneoformans isolated in Seville. *Spain. Mycoses* 60 40–50. 10.1111/myc.12543 27633849

[B29] Hagen F. Khayhan K. Theelen B. Kolecka A. Polacheck I. Sionov E. (2015). Recognition of seven species in the Cryptococcus gattii/Cryptococcus neoformans species complex. *Fungal. Genet. Biol.* 78 16–48. 10.1016/j.fgb.2015.02.009 25721988

[B30] Hagen F. Lumbsch H. T. Arsic Arsenijevic V. Badali H. Bertout S. Billmyre R. B. (2017). Importance of Resolving Fungal Nomenclature: the Case of Multiple Pathogenic Species in the Cryptococcus Genus. *mSphere* 2:e00238-17. 10.1128/mSphere.00238-17 28875175 PMC5577652

[B31] Hong N. Chen M. Xu J. (2021). Molecular Markers Reveal Epidemiological Patterns and Evolutionary Histories of the Human Pathogenic Cryptococcus. *Front. Cell. Infect. Microbiol.* 11:683670. 10.3389/fcimb.2021.683670 34026667 PMC8134695

[B32] Janbon G. Ormerod K. L. Paulet D. Byrnes E. J. III Yadav V. Chatterjee G. (2014). Analysis of the Genome and Transcriptome of Cryptococcus neoformans var. grubii Reveals Complex RNA Expression and Microevolution Leading to Virulence Attenuation. *PLoS Genet.* 10:e1004261. 10.1371/journal.pgen.1004261 24743168 PMC3990503

[B33] Khayhan K. Hagen F. Pan W. Simwami S. Fisher M. C. Wahyuningsih R. (2013). Geographically structured populations of Cryptococcus neoformans Variety grubii in Asia correlate with HIV status and show a clonal population structure. *PLoS One* 8:e72222. 10.1371/journal.pone.0072222 24019866 PMC3760895

[B34] Kwon-Chung K. J. Bennett J. E. Wickes B. L. Meyer W. Cuomo C. A. Wollenburg K. R. (2017). The Case for Adopting the “Species Complex” Nomenclature for the Etiologic Agents of Cryptococcosis. *mSphere* 2:e00357-16. 10.1128/mSphere.00357-16 28101535 PMC5227069

[B35] Li H. Durbin R. (2009). Fast and accurate short read alignment with Burrows–Wheeler transform. *Bioinformatics* 25 1754–1760. 10.1093/bioinformatics/btp324 19451168 PMC2705234

[B36] Li H. Handsaker B. Wysoker A. Fennell T. Ruan J. Homer N. (2009). The Sequence Alignment/Map format and SAMtools. *Bioinformatics* 25 2078–2079. 10.1093/bioinformatics/btp352 19505943 PMC2723002

[B37] Li M. Chen M. Pan W. (2013). Approaches on genetic polymorphism of Cryptococcus species complex. *Front. Biosci.* 18:1227–1236. 10.2741/4174 23747878

[B38] Li M. Liao Y. Chen M. Pan W. Weng L. (2012). Antifungal susceptibilities of Cryptococcus species complex isolates from AIDS and non-AIDS patients in Southeast China. *Braz. J. Infect. Dis.* 16 175–179. 10.1016/s1413-8670(12)70301-x 22552461

[B39] Li Y. Zou M. Yin J. Liu Z. Lu B. (2020). Microbiological, Epidemiological, and Clinical Characteristics of Patients With Cryptococcal Meningitis at a Tertiary Hospital in China: A 6-Year Retrospective Analysis. *Front. Microbiol.* 11:1837. 10.3389/fmicb.2020.01837 32849436 PMC7403485

[B40] McKenna A. Hanna M. Banks E. Sivachenko A. Cibulskis K. Kernytsky A. (2010). The Genome Analysis Toolkit: a MapReduce framework for analyzing next-generation DNA sequencing data. *Genome Res.* 20 1297–1303. 10.1101/gr.107524.110 20644199 PMC2928508

[B41] Meyer W. Aanensen D. M. Boekhout T. Cogliati M. Diaz M. R. Esposto M. C. (2009). Consensus multi-locus sequence typing scheme for Cryptococcus neoformans and Cryptococcus gattii. *Med. Mycol.* 47 561–570. 10.1080/13693780902953886 19462334 PMC2884100

[B42] Nguyen L. T. Schmidt H. A. von Haeseler A. Minh B. Q. (2015). IQ-TREE: a fast and effective stochastic algorithm for estimating maximum-likelihood phylogenies. *Mol. Biol. Evol.* 32 268–274. 10.1093/molbev/msu300 25371430 PMC4271533

[B43] Park B. J. Wannemuehler K. A. Marston B. J. Govender N. Pappas P. G. Chiller T. M. (2009). Estimation of the current global burden of cryptococcal meningitis among persons living with HIV/AIDS. *Aids* 23 525–530. 10.1097/QAD.0b013e328322ffac 19182676

[B44] Pyrgos V. Seitz A. E. Steiner C. A. Prevots D. R. Williamson P. R. (2013). Epidemiology of cryptococcal meningitis in the US: 1997-2009. *PLoS One* 8:e56269. 10.1371/journal.pone.0056269 23457543 PMC3574138

[B45] Rajasingham R. Smith R. M. Park B. J. Jarvis J. N. Govender N. P. Chiller T. M. (2017). Global burden of disease of HIV-associated cryptococcal meningitis: an updated analysis. *Lancet Infect. Dis.* 17 873–881. 10.1016/s1473-3099(17)30243-8 28483415 PMC5818156

[B46] Rhodes J. Desjardins C. A. Sykes S. M. Beale M. A. Vanhove M. Sakthikumar S. (2017). Tracing Genetic Exchange and Biogeography of Cryptococcus neoformans var. grubii at the Global Population Level. *Genetics* 207 327–346. 10.1534/genetics.117.203836 28679543 PMC5586382

[B47] Rodero L. Mellado E. Rodriguez A. C. Salve A. Guelfand L. Cahn P. (2003). G484S amino acid substitution in lanosterol 14-alpha demethylase (ERG11) is related to fluconazole resistance in a recurrent Cryptococcus neoformans clinical isolate. *Antimicrob. Agents Chemother.* 47 3653–3656. 10.1128/aac.47.11.3653-3656.2003 14576140 PMC253790

[B48] Selb R. Fuchs V. Graf B. Hamprecht A. Hogardt M. Sedlacek L. (2019). Molecular typing and *in vitro* resistance of Cryptococcus neoformans clinical isolates obtained in Germany between 2011 and 2017. *Int. J. Med. Microbiol.* 309:151336. 10.1016/j.ijmm.2019.151336 31444102

[B49] Sloan D. J. Parris V. (2014). Cryptococcal meningitis: epidemiology and therapeutic options. *Clin. Epidemiol.* 6 169–182. 10.2147/clep.S38850 24872723 PMC4026566

[B50] Stajich J. E. Harris T. Brunk B. P. Brestelli J. Fischer S. Harb O. S. (2012). FungiDB: an integrated functional genomics database for fungi. *Nucleic Acids Res.* 40:D675–D681. 10.1093/nar/gkr918 22064857 PMC3245123

[B51] Stone N. R. Rhodes J. Fisher M. C. Mfinanga S. Kivuyo S. Rugemalila J. (2019). Dynamic ploidy changes drive fluconazole resistance in human cryptococcal meningitis. *J. Clin. Invest.* 129 999–1014. 10.1172/jci124516 30688656 PMC6391087

[B52] Thanh L. T. Phan T. H. Rattanavong S. Nguyen T. M. Duong A. V. Dacon C. (2018). Multilocus sequence typing of Cryptococcus neoformans var. grubii from Laos in a regional and global context. *Med. Mycol.* 57 557–565. 10.1093/mmy/myy105 30339200 PMC6581559

[B53] Thorvaldsdóttir H. Robinson J. T. Mesirov J. P. (2013). Integrative Genomics Viewer (IGV): high-performance genomics data visualization and exploration. *Brief. Bioinformatics* 14 178–192. 10.1093/bib/bbs017 22517427 PMC3603213

[B54] Vu K. Thompson G. R. III Roe C. C. Sykes J. E. Dreibe E. M. Lockhart S. R. (2018). Flucytosine resistance in Cryptococcus gattii is indirectly mediated by the FCY2-FCY1-FUR1 pathway. *Med. Mycol.* 56 857–867. 10.1093/mmy/myx135 29554336 PMC10905989

[B55] Wu S. Y. Kang M. Liu Y. Chen Z. X. Xiao Y. L. He C. (2021). Molecular epidemiology and antifungal susceptibilities of Cryptococcus species isolates from HIV and non-HIV patients in Southwest China. *Eur. J. Clin. Microbiol. Infect. Dis.* 40 287–295. 10.1007/s10096-020-04013-4 32895755

[B56] Xess I. Pandey M. Dabas Y. Agarwal R. Das S. Srivastava P. M. V. (2021). Multilocus Sequence Typing of Clinical Isolates of Cryptococcus from India. *Mycopathologia* 186 199–211. 10.1007/s11046-020-00500-6 33469844

[B57] Xiao M. Chen S. C. Kong F. Fan X. Cheng J. W. Hou X. (2018). Five-year China Hospital Invasive Fungal Surveillance Net (CHIF-NET) study of invasive fungal infections caused by noncandidal yeasts: species distribution and azole susceptibility. *Infect. Drug. Resist.* 11 1659–1667. 10.2147/idr.S173805 30349323 PMC6183553

[B58] Xu J. Ramos A. R. Vilgalys R. Mitchell T. G. (2000). Clonal and spontaneous origins of fluconazole resistance in Candida albicans. *J. Clin. Microbiol.* 38 1214–1220. 10.1128/jcm.38.3.1214-1220.2000 10699025 PMC86380

[B59] Yang C. Bian Z. Blechert O. Deng F. Chen H. Li Y. (2021). High Prevalence of HIV-Related Cryptococcosis and Increased Resistance to Fluconazole of the Cryptococcus neoformans Complex in Jiangxi Province. South Central China. *Front. Cell. Infect. Microbiol.* 11:723251. 10.3389/fcimb.2021.723251 34790585 PMC8592285

[B60] Yang F. Gritsenko V. Lu H. Zhen C. Gao L. Berman J. (2021). Adaptation to Fluconazole via Aneuploidy Enables Cross-Adaptation to Amphotericin B and Flucytosine in Cryptococcus neoformans. *Microbiol. Spectr.* 9:e0072321. 10.1128/Spectrum.00723-21 34585947 PMC8557924

