# Supplementary material for: Molecular Epidemiology and Antifungal Resistance of Cryptococcus neoformans From Human Immunodeficiency Virus-Negative and Human Immunodeficiency Virus-Positive Patients in Eastern China
Source: Front Microbiol. 2022 Jul 5;13:942940. doi: 10.3389/fmicb.2022.942940 (PMC9294546; doi:10.3389/fmicb.2022.942940)
Supplement: Supplementary file 1 [file Table_1.DOCX]

Table S1. MIC distributions of *Cryptococcus neoformans* against four antifungal drugs

| HIV status | Species (No. of isolates) | Antifungal drugs | MIC (μg/mL) | | | | | | | | | | | | |
| --- | --- | --- | --- | --- | --- | --- | --- | --- | --- | --- | --- | --- | --- | --- | --- |
|  |  |  | 64 | 32 | 16 | 8 | 4 | 2 | 1 | 0.5 | 0.25 | 0.125 | 0.0625 | 0.03125 | 0.015 |
| HIV-positive | *Cryptococcus neoformans* (n=61) | Fluconazole |  | 1 | 2 | 18 | 26 | 12 | 2 |  |  |  |  |  |  |
|  |  | Voriconazole |  |  |  |  |  |  |  |  | 3 | 23 | 28 | 7 |  |
|  |  | Amphotericin B |  |  |  |  |  | 26 | 29 | 4 | 2 |  |  |  |  |
|  |  | Flucytosine |  |  | 1 | 15 | 33 | 11 | 1 |  |  |  |  |  |  |
| HIV-negative | *Cryptococcus neoformans* (n=72) | Fluconazole |  |  | 5 | 24 | 32 | 10 | 1 |  |  |  |  |  |  |
|  |  | Voriconazole |  |  |  |  |  |  |  |  | 7 | 23 | 34 | 7 | 1 |
|  |  | Amphotericin B |  |  |  |  |  | 27 | 27 | 11 | 6 | 1 |  |  |  |
|  |  | Flucytosine | 3 | 3 | 1 | 32 | 30 | 3 |  |  |  |  |  |  |  |

MIC, minimum inhibitory concentration.
