# Supplementary material for: Molecular Epidemiology and Antifungal Resistance of Cryptococcus neoformans From Human Immunodeficiency Virus-Negative and Human Immunodeficiency Virus-Positive Patients in Eastern China
Source: Front Microbiol. 2022 Jul 5;13:942940. doi: 10.3389/fmicb.2022.942940 (PMC9294546; doi:10.3389/fmicb.2022.942940)
Supplement: Supplementary file 2 [file Table_2.DOCX]

Table S2. Sequence type of Cryptococcus neoformans species from HIV-positive patients

| ID | Molecular type | Sequence Type | CAP59 | GPD1 | IGS1 | PLB1 | SOD1 | URA5 | LAC1 |
| --- | --- | --- | --- | --- | --- | --- | --- | --- | --- |
| YQJ010 | VNI | ST5 | AT1 | AT3 | AT1 | AT2 | AT1 | AT1 | AT5 |
| YQJ016 | VNI | ST5 | AT1 | AT3 | AT1 | AT2 | AT1 | AT1 | AT5 |
| YQJ017 | VNI | ST5 | AT1 | AT3 | AT1 | AT2 | AT1 | AT1 | AT5 |
| YQJ018 | VNI | ST5 | AT1 | AT3 | AT1 | AT2 | AT1 | AT1 | AT5 |
| YQJ019 | VNI | ST5 | AT1 | AT3 | AT1 | AT2 | AT1 | AT1 | AT5 |
| YQJ020 | VNI | ST5 | AT1 | AT3 | AT1 | AT2 | AT1 | AT1 | AT5 |
| YQJ167 | VNI | ST5 | AT1 | AT3 | AT1 | AT2 | AT1 | AT1 | AT5 |
| YQJ168 | VNI | ST5 | AT1 | AT3 | AT1 | AT2 | AT1 | AT1 | AT5 |
| YQJ169 | VNI | ST5 | AT1 | AT3 | AT1 | AT2 | AT1 | AT1 | AT5 |
| YQJ170 | VNI | ST5 | AT1 | AT3 | AT1 | AT2 | AT1 | AT1 | AT5 |
| YQJ171 | VNI | ST5 | AT1 | AT3 | AT1 | AT2 | AT1 | AT1 | AT5 |
| YQJ172 | VNI | ST5 | AT1 | AT3 | AT1 | AT2 | AT1 | AT1 | AT5 |
| YQJ175 | VNII | ST43 | AT2 | AT9 | AT14 | AT11 | AT11 | AT4 | AT8 |
| YQJ176 | VNI | ST93 | AT1 | AT23 | AT10 | AT4 | AT1 | AT1 | AT3 |
| YQJ185 | VNI | ST5 | AT1 | AT3 | AT1 | AT2 | AT1 | AT1 | AT5 |
| YQJ190 | VNI | ST5 | AT1 | AT3 | AT1 | AT2 | AT1 | AT1 | AT5 |
| YQJ194 | VNI | ST5 | AT1 | AT3 | AT1 | AT2 | AT1 | AT1 | AT5 |
| YQJ205 |  |  | AT7 |  | AT1 | AT1 | AT1 | AT1 |  |
| YQJ208 | VNI | ST5 | AT1 | AT3 | AT1 | AT2 | AT1 | AT1 | AT5 |
| YQJ209 |  |  | AT7 | AT1 | AT1 | AT1 | AT1 | AT1 |  |
| YQJ219 | VNI | ST5 | AT1 | AT3 | AT1 | AT2 | AT1 | AT1 | AT5 |
| YQJ220 | VNI | ST5 | AT1 | AT3 | AT1 | AT2 | AT1 | AT1 | AT5 |
| YQJ221 |  |  | AT7 | AT1 | AT1 | AT1 | AT1 | AT1 |  |
| YQJ233 | VNI | ST5 | AT1 | AT3 | AT1 | AT2 | AT1 | AT1 | AT5 |
| YQJ236 | VNI | ST5 | AT1 | AT3 | AT1 | AT2 | AT1 | AT1 | AT5 |
| YQJ246 | VNI | ST5 | AT1 | AT3 | AT1 | AT2 | AT1 | AT1 | AT5 |
| YQJ249 | VNI | ST77 | AT1 | AT1 | AT25 | AT2 | AT1 | AT1 | AT3 |
| YQJ261 | VNI | ST5 | AT1 | AT3 | AT1 | AT2 | AT1 | AT1 | AT5 |
| YQJ262 | VNI | ST5 | AT1 | AT3 | AT1 | AT2 | AT1 | AT1 | AT5 |
| YQJ263 | VNI | ST5 | AT1 | AT3 | AT1 | AT2 | AT1 | AT1 | AT5 |
| YQJ290 | VNI | ST5 | AT1 | AT3 | AT1 | AT2 | AT1 | AT1 | AT5 |
| YQJ305 | VNI | ST63 | AT7 | AT1 | AT1 | AT1 | AT1 | AT1 | AT18 |
| YQJ306 | VNI | ST230 | AT7 | AT1 | AT1 | AT1 | AT1 | AT1 | AT5 |
| YQJ307 | VNI | ST5 | AT1 | AT3 | AT1 | AT2 | AT1 | AT1 | AT5 |
| YQJ308 | VNI | ST5 | AT1 | AT3 | AT1 | AT2 | AT1 | AT1 | AT5 |
| YQJ309 | VNI | ST5 | AT1 | AT3 | AT1 | AT2 | AT1 | AT1 | AT5 |
| YQJ313 | VNI | ST5 | AT1 | AT3 | AT1 | AT2 | AT1 | AT1 | AT5 |
| YQJ314 | VNI | ST5 | AT1 | AT3 | AT1 | AT2 | AT1 | AT1 | AT5 |
| YQJ315 | VNI | ST5 | AT1 | AT3 | AT1 | AT2 | AT1 | AT1 | AT5 |
| YQJ316 | VNI | ST5 | AT1 | AT3 | AT1 | AT2 | AT1 | AT1 | AT5 |
| YQJ317 | VNI | ST5 | AT1 | AT3 | AT1 | AT2 | AT1 | AT1 | AT5 |
| YQJ318 | VNI | ST5 | AT1 | AT3 | AT1 | AT2 | AT1 | AT1 | AT5 |
| YQJ319 | VNI | ST5 | AT1 | AT3 | AT1 | AT2 | AT1 | AT1 | AT5 |
| YQJ320 | VNI | ST5 | AT1 | AT3 | AT1 | AT2 | AT1 | AT1 | AT5 |
| YQJ322 | VNI | ST5 | AT1 | AT3 | AT1 | AT2 | AT1 | AT1 | AT5 |
| YQJ323 | VNI | ST5 | AT1 | AT3 | AT1 | AT2 | AT1 | AT1 | AT5 |
| YQJ324 | VNI | ST5 | AT1 | AT3 | AT1 | AT2 | AT1 | AT1 | AT5 |
| YQJ325 | VNI | ST5 | AT1 | AT3 | AT1 | AT2 | AT1 | AT1 | AT5 |
| YQJ328 | VNI | ST5 | AT1 | AT3 | AT1 | AT2 | AT1 | AT1 | AT5 |
| YQJ329 | VNI | ST5 | AT1 | AT3 | AT1 | AT2 | AT1 | AT1 | AT5 |
| YQJ331 | VNI | ST5 | AT1 | AT3 | AT1 | AT2 | AT1 | AT1 | AT5 |
| YQJ332 | VNI | ST5 | AT1 | AT3 | AT1 | AT2 | AT1 | AT1 | AT5 |
| YQJ333 | VNI | ST5 | AT1 | AT3 | AT1 | AT2 | AT1 | AT1 | AT5 |
| YQJ334 | VNI | ST5 | AT1 | AT3 | AT1 | AT2 | AT1 | AT1 | AT5 |
| YQJ335 | VNI | ST5 | AT1 | AT3 | AT1 | AT2 | AT1 | AT1 | AT5 |
| YQJ337 | VNI | ST5 | AT1 | AT3 | AT1 | AT2 | AT1 | AT1 | AT5 |
| YQJ341 | VNI | ST5 | AT1 | AT3 | AT1 | AT2 | AT1 | AT1 | AT5 |
| YQJ348 | VNI | ST5 | AT1 | AT3 | AT1 | AT2 | AT1 | AT1 | AT5 |
| YQJ351 | VNI | ST5 | AT1 | AT3 | AT1 | AT2 | AT1 | AT1 | AT5 |
| YQJ352 | VNI | ST5 | AT1 | AT3 | AT1 | AT2 | AT1 | AT1 | AT5 |
| YQJ353 | VNI | ST5 | AT1 | AT3 | AT1 | AT2 | AT1 | AT1 | AT5 |
