# Supplementary material for: Molecular Epidemiology and Antifungal Resistance of Cryptococcus neoformans From Human Immunodeficiency Virus-Negative and Human Immunodeficiency Virus-Positive Patients in Eastern China
Source: Front Microbiol. 2022 Jul 5;13:942940. doi: 10.3389/fmicb.2022.942940 (PMC9294546; doi:10.3389/fmicb.2022.942940)
Supplement: Supplementary file 3 [file Table_3.DOCX]

Table S3. Sequence type of Cryptococcus neoformans species from HIV-negative patients

| ID | Molecular type | Sequence Type | CAP59 | GPD1 | IGS1 | PLB1 | SOD1 | URA5 | LAC1 |
| --- | --- | --- | --- | --- | --- | --- | --- | --- | --- |
| YQJ173 | VNI | ST5 | AT1 | AT3 | AT1 | AT2 | AT1 | AT1 | AT5 |
| YQJ177 | VNI | ST5 | AT1 | AT3 | AT1 | AT2 | AT1 | AT1 | AT5 |
| YQJ178 | VNI | ST5 | AT1 | AT3 | AT1 | AT2 | AT1 | AT1 | AT5 |
| YQJ179 | VNI | ST5 | AT1 | AT3 | AT1 | AT2 | AT1 | AT1 | AT5 |
| YQJ180 | VNI | ST5 | AT1 | AT3 | AT1 | AT2 | AT1 | AT1 | AT5 |
| YQJ181 | VNI | ST5 | AT1 | AT3 | AT1 | AT2 | AT1 | AT1 | AT5 |
| YQJ182 | VNI | ST5 | AT1 | AT3 | AT1 | AT2 | AT1 | AT1 | AT5 |
| YQJ183 | VNI | ST5 | AT1 | AT3 | AT1 | AT2 | AT1 | AT1 | AT5 |
| YQJ184 | VNI | ST5 | AT1 | AT3 | AT1 | AT2 | AT1 | AT1 | AT5 |
| YQJ186 | VNI | ST5 | AT1 | AT3 | AT1 | AT2 | AT1 | AT1 | AT5 |
| YQJ187 | VNI | ST5 | AT1 | AT3 | AT1 | AT2 | AT1 | AT1 | AT5 |
| YQJ188 | VNI | ST5 | AT1 | AT3 | AT1 | AT2 | AT1 | AT1 | AT5 |
| YQJ191 | VNI | ST5 | AT1 | AT3 | AT1 | AT2 | AT1 | AT1 | AT5 |
| YQJ192 | VNI | ST5 | AT1 | AT3 | AT1 | AT2 | AT1 | AT1 | AT5 |
| YQJ195 | VNI | ST5 | AT1 | AT3 | AT1 | AT2 | AT1 | AT1 | AT5 |
| YQJ196 | VNI | ST185 | AT1 | AT1 | AT10 | AT2 | AT36 | AT1 | AT3 |
| YQJ197 | VNI | ST5 | AT1 | AT3 | AT1 | AT2 | AT1 | AT1 | AT5 |
| YQJ198 | VNI | ST5 | AT1 | AT3 | AT1 | AT2 | AT1 | AT1 | AT5 |
| YQJ228 | VNI | ST5 | AT1 | AT3 | AT1 | AT2 | AT1 | AT1 | AT5 |
| YQJ229 | VNI | ST5 | AT1 | AT3 | AT1 | AT2 | AT1 | AT1 | AT5 |
| YQJ230 | VNI | ST5 | AT1 | AT3 | AT1 | AT2 | AT1 | AT1 | AT5 |
| YQJ231 | VNI | ST5 | AT1 | AT3 | AT1 | AT2 | AT1 | AT1 | AT5 |
| YQJ232 | VNI | ST5 | AT1 | AT3 | AT1 | AT2 | AT1 | AT1 | AT5 |
| YQJ235 | VNI | ST5 | AT1 | AT3 | AT1 | AT2 | AT1 | AT1 | AT5 |
| YQJ237 | VNI | ST5 | AT1 | AT3 | AT1 | AT2 | AT1 | AT1 | AT5 |
| YQJ241 | VNI | ST5 | AT1 | AT3 | AT1 | AT2 | AT1 | AT1 | AT5 |
| YQJ242 | VNI | ST5 | AT1 | AT3 | AT1 | AT2 | AT1 | AT1 | AT5 |
| YQJ243 | VNI | ST5 | AT1 | AT3 | AT1 | AT2 | AT1 | AT1 | AT5 |
| YQJ244 | VNI | ST5 | AT1 | AT3 | AT1 | AT2 | AT1 | AT1 | AT5 |
| YQJ245 | VNI | ST5 | AT1 | AT3 | AT1 | AT2 | AT1 | AT1 | AT5 |
| YQJ248 | VNI | ST5 | AT1 | AT3 | AT1 | AT2 | AT1 | AT1 | AT5 |
| YQJ251 | VNI | ST5 | AT1 | AT3 | AT1 | AT2 | AT1 | AT1 | AT5 |
| YQJ252 | VNI | ST5 | AT1 | AT3 | AT1 | AT2 | AT1 | AT1 | AT5 |
| YQJ253 | VNI | ST31 | AT1 | AT1 | AT10 | AT2 | AT1 | AT1 | AT3 |
| YQJ255 | VNI | ST5 | AT1 | AT3 | AT1 | AT2 | AT1 | AT1 | AT5 |
| YQJ256 | VNI | ST5 | AT1 | AT3 | AT1 | AT2 | AT1 | AT1 | AT5 |
| YQJ257 | VNI | ST5 | AT1 | AT3 | AT1 | AT2 | AT1 | AT1 | AT5 |
| YQJ258 | VNI | ST5 | AT1 | AT3 | AT1 | AT2 | AT1 | AT1 | AT5 |
| YQJ260 | VNI | ST5 | AT1 | AT3 | AT1 | AT2 | AT1 | AT1 | AT5 |
| YQJ011 | VNI | ST5 | AT1 | AT3 | AT1 | AT2 | AT1 | AT1 | AT5 |
| YQJ057 | VNI | ST5 | AT1 | AT3 | AT1 | AT2 | AT1 | AT1 | AT5 |
| YQJ174 | VNI | ST5 | AT1 | AT3 | AT1 | AT2 | AT1 | AT1 | AT5 |
| YQJ194 | VNI | ST5 | AT1 | AT3 | AT1 | AT2 | AT1 | AT1 | AT5 |
| YQJ201 | VNI | ST5 | AT1 | AT3 | AT1 | AT2 | AT1 | AT1 | AT5 |
| YQJ067 | VNI | ST5 | AT1 | AT3 | AT1 | AT2 | AT1 | AT1 | AT5 |
| YQJ068 | VNI | ST5 | AT1 | AT3 | AT1 | AT2 | AT1 | AT1 | AT5 |
| YQJ144 |  |  |  |  | AT1 |  |  |  |  |
| YQJ226 | VNI | ST5 | AT1 | AT3 | AT1 | AT2 | AT1 | AT1 | AT5 |
| YQJ227 | VNI | ST5 | AT1 | AT3 | AT1 | AT2 | AT1 | AT1 | AT5 |
| YQJ247 | VNI | ST5 | AT1 | AT3 | AT1 | AT2 | AT1 | AT1 | AT5 |
| YQJ272 | VNI | ST5 | AT1 | AT3 | AT1 | AT2 | AT1 | AT1 | AT5 |
| YQJ303 | VNI | ST5 | AT1 | AT3 | AT1 | AT2 | AT1 | AT1 | AT5 |
| YQJ304 | VNI | ST5 | AT1 | AT3 | AT1 | AT2 | AT1 | AT1 | AT5 |
| YQJ310 | VNI | ST5 | AT1 | AT3 | AT1 | AT2 | AT1 | AT1 | AT5 |
| YQJ311 | VNI | ST5 | AT1 | AT3 | AT1 | AT2 | AT1 | AT1 | AT5 |
| YQJ312 | VNI | ST5 | AT1 | AT3 | AT1 | AT2 | AT1 | AT1 | AT5 |
| YQJ321 | VNI | ST5 | AT1 | AT3 | AT1 | AT2 | AT1 | AT1 | AT5 |
| YQJ326 | VNI | ST5 | AT1 | AT3 | AT1 | AT2 | AT1 | AT1 | AT5 |
| YQJ327 | VNI | ST5 | AT1 | AT3 | AT1 | AT2 | AT1 | AT1 | AT5 |
| YQJ330 | VNI | ST5 | AT1 | AT3 | AT1 | AT2 | AT1 | AT1 | AT5 |
| YQJ336 | VNI | ST5 | AT1 | AT3 | AT1 | AT2 | AT1 | AT1 | AT5 |
| YQJ338 | VNI | ST5 | AT1 | AT3 | AT1 | AT2 | AT1 | AT1 | AT5 |
| YQJ339 | VNI | ST653 | AT1 | AT3 | AT1 | AT43 | AT1 | AT1 | AT5 |
| YQJ340 | VNI | ST653 | AT1 | AT3 | AT1 | AT44 | AT1 | AT1 | AT5 |
| YQJ342 | VNI | ST5 | AT1 | AT3 | AT1 | AT2 | AT1 | AT1 | AT5 |
| YQJ343 | VNI | ST5 | AT1 | AT3 | AT1 | AT2 | AT1 | AT1 | AT5 |
| YQJ344 | VNI | ST5 | AT1 | AT3 | AT1 | AT2 | AT1 | AT1 | AT5 |
| YQJ345 | VNI | ST5 | AT1 | AT3 | AT1 | AT2 | AT1 | AT1 | AT5 |
| YQJ346 | VNI | ST5 | AT1 | AT3 | AT1 | AT2 | AT1 | AT1 | AT5 |
| YQJ347 | VNI | ST5 | AT1 | AT3 | AT1 | AT2 | AT1 | AT1 | AT5 |
| YQJ349 | VNI | ST5 | AT1 | AT3 | AT1 | AT2 | AT1 | AT1 | AT5 |
| YQJ350 | VNI | ST5 | AT1 | AT3 | AT1 | AT2 | AT1 | AT1 | AT5 |
