# Supplementary material for: Molecular Epidemiology and Antifungal Resistance of Cryptococcus neoformans From Human Immunodeficiency Virus-Negative and Human Immunodeficiency Virus-Positive Patients in Eastern China
Source: Front Microbiol. 2022 Jul 5;13:942940. doi: 10.3389/fmicb.2022.942940 (PMC9294546; doi:10.3389/fmicb.2022.942940)
Supplement: Supplementary file 4 [file Table_4.DOCX]

Table S4. Details of whole-genome sequencing of three multi-drug resistance isolates

| Sample ID | YQJ068 | YQJ185 | YQJ247 |
| --- | --- | --- | --- |
| Raw_total_reads | 10726596 | 8740232 | 8665542 |
| Raw_total_bases | 1615254780 | 1316135233 | 1304887358 |
| Total_reads_after_QC | 10345914 | 8457356 | 8395328 |
| Total_bases_after_QC | 1543085055 | 1258825937 | 1250903472 |
| Q20_rate_after_QC (%) | 91.9129 | 91.7229 | 91.8592 |
| Q30_rate_after_QC (%) | 86.0082 | 85.7386 | 85.9297 |
| Average_depth | 80.13 | 65.41 | 64.9 |
| Coverage (>0x) | 99.45% | 99.28% | 99.38% |
| Coverage (>=10x) | 98.81% | 98.53% | 98.64% |
