# Supplementary material for: Molecular Epidemiology and Antifungal Resistance of Cryptococcus neoformans From Human Immunodeficiency Virus-Negative and Human Immunodeficiency Virus-Positive Patients in Eastern China
Source: Front Microbiol. 2022 Jul 5;13:942940. doi: 10.3389/fmicb.2022.942940 (PMC9294546; doi:10.3389/fmicb.2022.942940)
Supplement: Supplementary file 5 [file Table_5.DOCX]

Table S5. Demographic Characteristics and potential diseases of HIV-infected and uninfected patients with cryptococcosis from China

| **Parameter** | **Total** | **No. HIV-infected** | **No. HIV-uninfected** |
| --- | --- | --- | --- |
| **Total** | 133 | 61 | 72 |
| **Gender** |  |  |  |
| Male | 93 | 43 | 50 |
| Female | 15 | 6 | 9 |
| Unknown states | 25 | 12 | 13 |
| **Ages(years)** |  |  |  |
| ≤14 | 1 | 0 | 1^*^ |
| 15-24 | 1 | 1 | 0 |
| 25-34 | 23 | 11 | 12 |
| 35-44 | 30 | 17 | 13 |
| 45-54 | 15 | 8 | 7 |
| 55-64 | 15 | 3 | 12 |
| ≥65 | 19 | 5 | 14 |
| Unknown states^**^ | 29 | 16 | 13 |
| **Risk factors** |  |  |  |
| Autoimmune diseases | / | 61 | 5 |
| Hepatitis and liver cirrhosis | / | / | 9 |
| Pneumonia | / | / | 7 |
| Tuberculosis | / | / | 2 |
| Organ transplantation | / | / | 3 |
| Renal diseases | / | / | 2 |
| Hematologic malignancy | / | / | 2 |
| Intracranial infection | / | / | 14 |
| Diabetes | / | / | 1 |
| Malignant tumor | / | / | 1 |
| other underlying diseases | / | / | 26^***^ |
| **Specimen** |  |  |  |
| Cerebrospinal fluid (CSF) | 84 | 49 | 35 |
| Blood | 25 | 10 | 15 |
| Hydrothorax | 6 | 2 | 4 |
| Ascites | 2 | / | 2 |
| Bile | 1 | / | 1 |
| Pulmonary tissue | 3 | / | 3 |
| Purulent secretion | 1 | / | 1 |
| Sputum | 1 | / | 1 |
| Urine | 1 | / | 1 |
| broncholveolr lvge fluid (BLF) | 1 | / | 1 |
| Unknown states | 8 | / | 8 |

* 2 years old; **Includes the middle-aged; ***Includes surgery, hypertension and unknown states.
