# Supplementary material for: Molecular Epidemiology and Antifungal Resistance of Cryptococcus neoformans From Human Immunodeficiency Virus-Negative and Human Immunodeficiency Virus-Positive Patients in Eastern China
Source: Front Microbiol. 2022 Jul 5;13:942940. doi: 10.3389/fmicb.2022.942940 (PMC9294546; doi:10.3389/fmicb.2022.942940)
Supplement: Supplementary file 6 [file Table_6.DOCX]

Table S6. Region and collection year of *C. neoformans*

|  | Region | | |
| --- | --- | --- | --- |
| year | Shanghai | Guangxi | Jiangsu |
| 2017 | 13 | 2 | 0 |
| 2018 | 75 | 8 | 1 |
| 2019 | 14 | 0 | 0 |
| 2020 | 20 | 0 | 0 |
